# Supplementary figures and images for: A Multitrait Locus Regulates Sarbecovirus Pathogenesis
Source: mBio. 2022 Jul 12;13(4):e01454-22. doi: 10.1128/mbio.01454-22 (PMC9426612; doi:10.1128/mbio.01454-22)

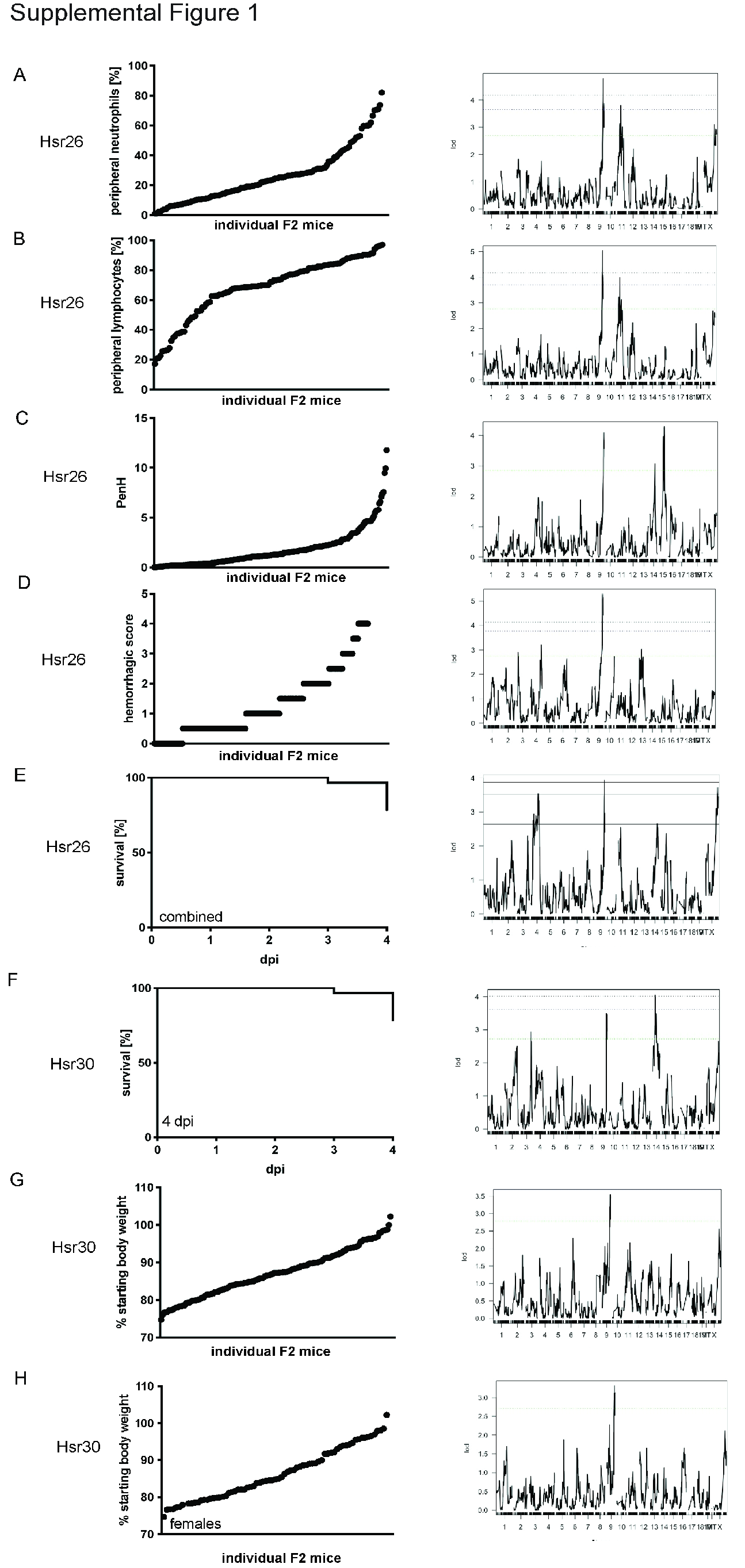

Supplement: FIG S1 [file mbio.01454-22-s0005.tif]

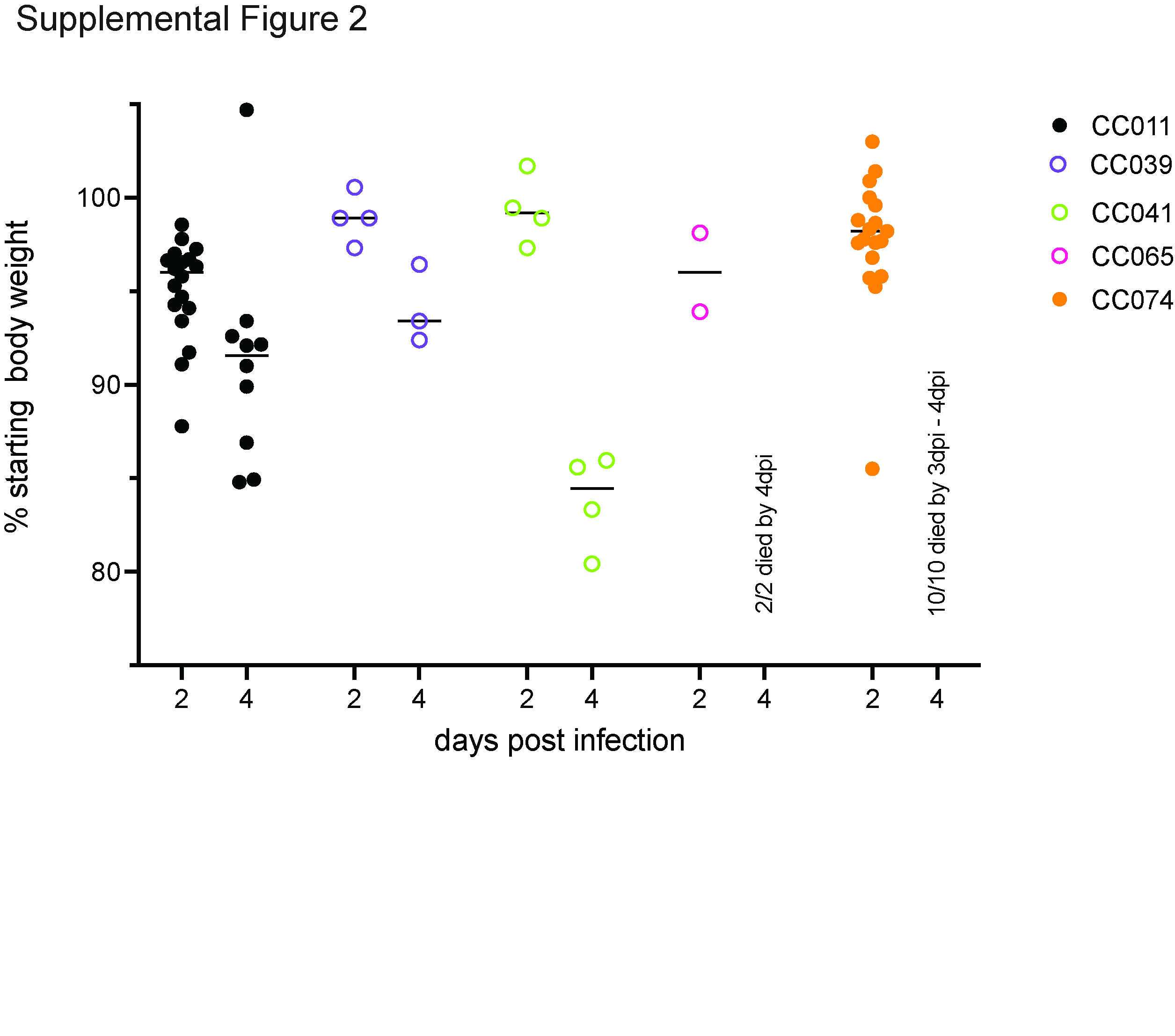

Supplement: FIG S2 [file mbio.01454-22-s0002.tif]

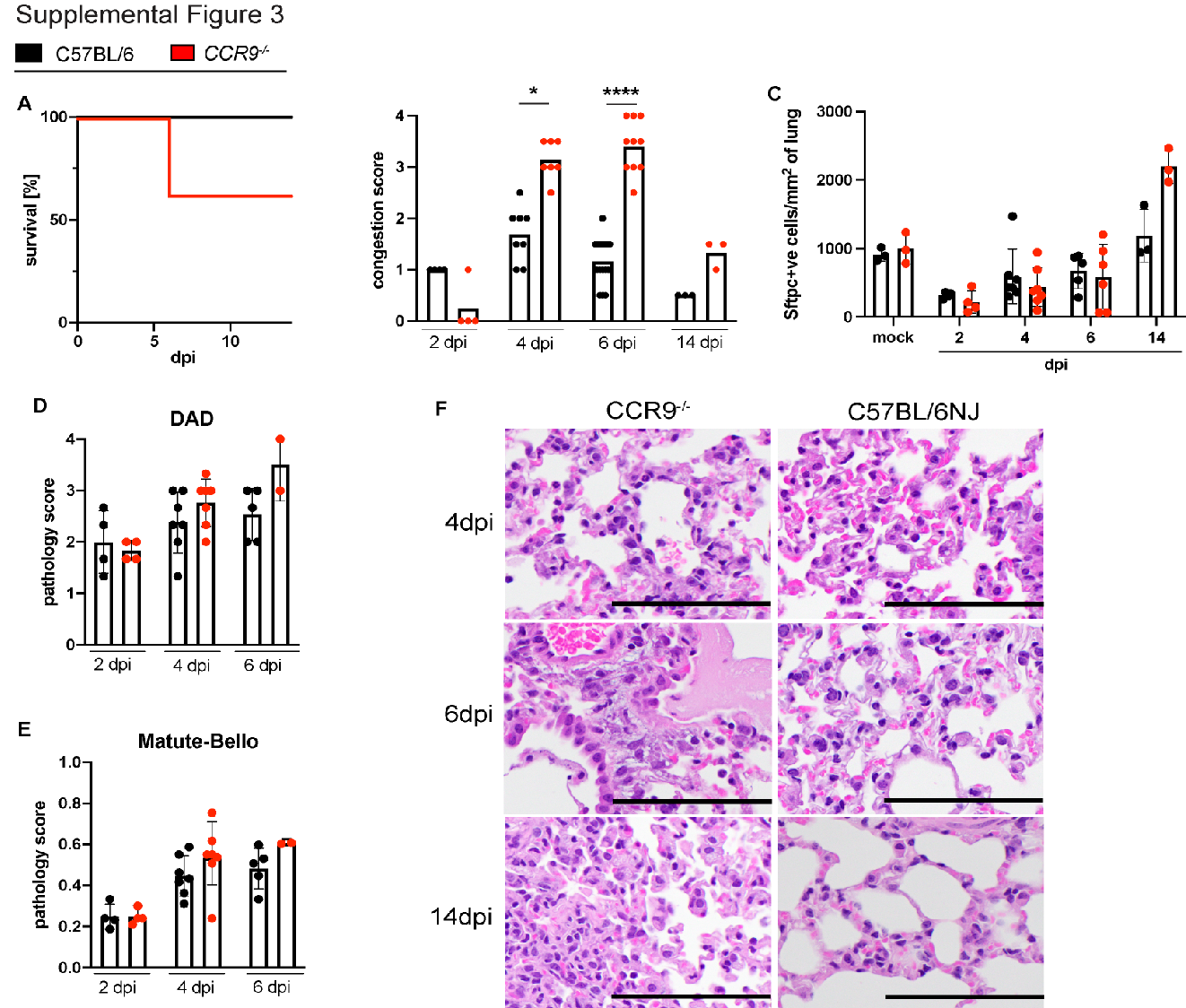

Supplement: FIG S3 [file mbio.01454-22-s0006.tif]

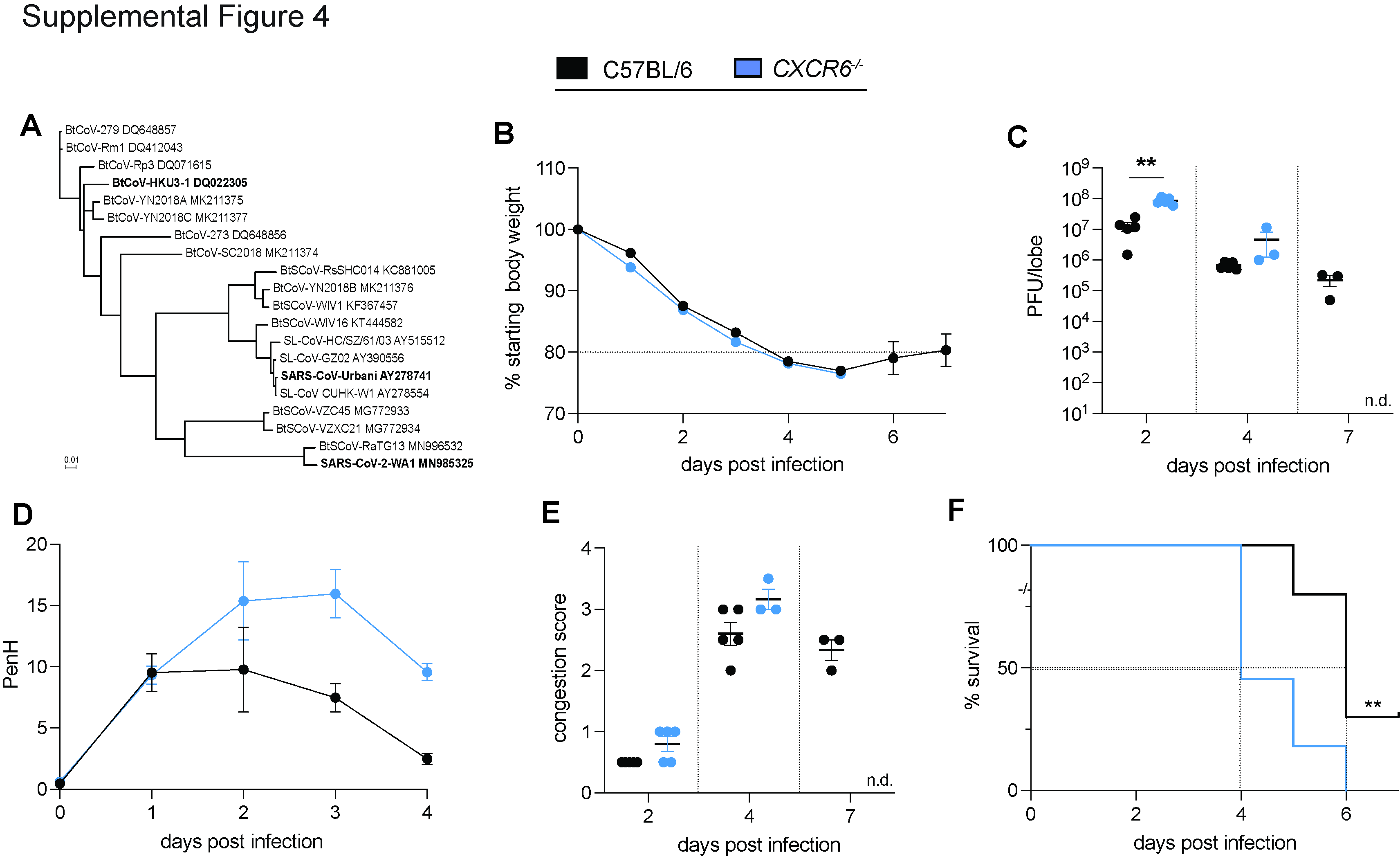

Supplement: FIG S4 [file mbio.01454-22-s0003.tif]

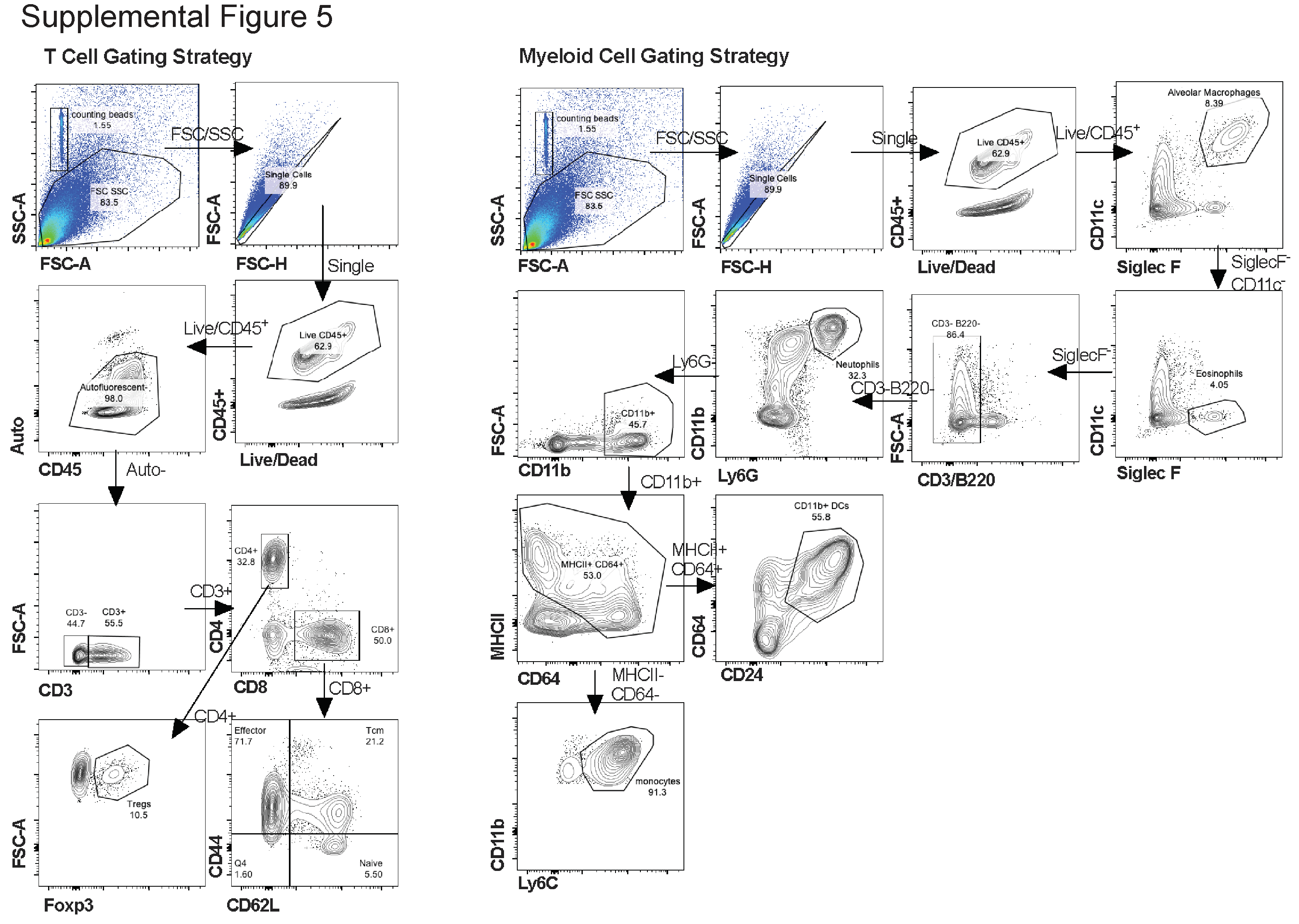

Supplement: FIG S5 [file mbio.01454-22-s0001.tif]
