# Supplementary material for: A Multitrait Locus Regulates Sarbecovirus Pathogenesis
Source: mBio. 2022 Jul 12;13(4):e01454-22. doi: 10.1128/mbio.01454-22 (PMC9426612; doi:10.1128/mbio.01454-22)
Supplement: TABLE S1 [file mbio.01454-22-s0004.pdf]

Table S1

Basal_Seq (2)

Strain	Class	CCR9.Liv	CCR9.Kid	CCR9.Ht	CXCR6.Liv	CXCR6.Kid	CXCR6.Ht	XCR1.Liv	XCR1.Kid	XCR1.Ht	LZTFL1.Liv	LZTFL1.Kid	LZTFL1.Ht	FYCO1.Liv	FYCO1.Kid	FYCO1.Ht	SLC6A20B.Liv	SLC6A20B.Kid	SLC6A20B.Ht
**CC001**	p	5.0942	4.7871	5.8392	5.349	4.739	5.9672	4.8839	4.8259	5.6648	8.5261	9.4575	9.0498	11.2655	11.1361	13.5345	4.6917	11.5407	5.1919
**CC011**	p	5.0016	5.1099	7.1759	4.438	4.7545	5.849	4.6836	4.5831	5.423	8.4163	9.5128	9.2282	11.2307	11.1413	13.7246	4.438	12.024	5.1919
**CC015**	p	4.8306	4.6154	5.7753	4.663	5.0498	6.0737	5.2146	4.7052	6.1117	8.3525	9.5224	9.1634	11.6362	11.3682	13.5997	4.4727	11.9893	5.7041
**CC025**	p	5.1712	4.9381	5.6846	4.9427	4.6773	5.5162	5.0924	4.7954	5.7694	8.2529	9.4876	9.1442	11.206	11.2029	13.6806	4.4999	11.9355	5.5759
**CC029**	a	4.764	4.6352	5.938	4.8046	4.3202	5.6199	4.7478	4.9505	6.0327	8.4996	9.7361	9.6589	11.0995	10.7345	14.0004	4.4122	12.6124	5.7151
**CC045**	a	4.953	4.8883	5.5524	5.2101	4.9223	5.6582	5.2178	5.0154	5.7795	8.2331	10.0202	9.6293	10.8453	11.0464	13.8474	4.5025	12.9219	5.4956
**CC051**	a	4.8261	4.7771	7.9224	5.287	4.9332	6.0919	5.0173	4.7536	6.3416	8.5401	9.7245	9.5628	10.9689	10.6005	13.7416	4.2537	12.3666	5.5739
**CC059**	a	5.3141	4.7684	6.9889	5.8097	5.5077	6.2412	4.9692	5.2876	6.0868	8.1732	9.8995	9.5278	10.9814	10.9644	13.7105	4.4522	12.5108	5.5183
